# Supplementary material for: Plant Cuticles Exhibit Significant Mid-Infrared Emissivity in the Atmospheric Windows
Source: Int J Mol Sci. 2025 Oct 12;26(20):9917. doi: 10.3390/ijms26209917 (PMC12562811; doi:10.3390/ijms26209917)
Supplement: Supplementary file 1 [file ijms-26-09917-s001.zip › ijms-3890944-supplementary.pdf]

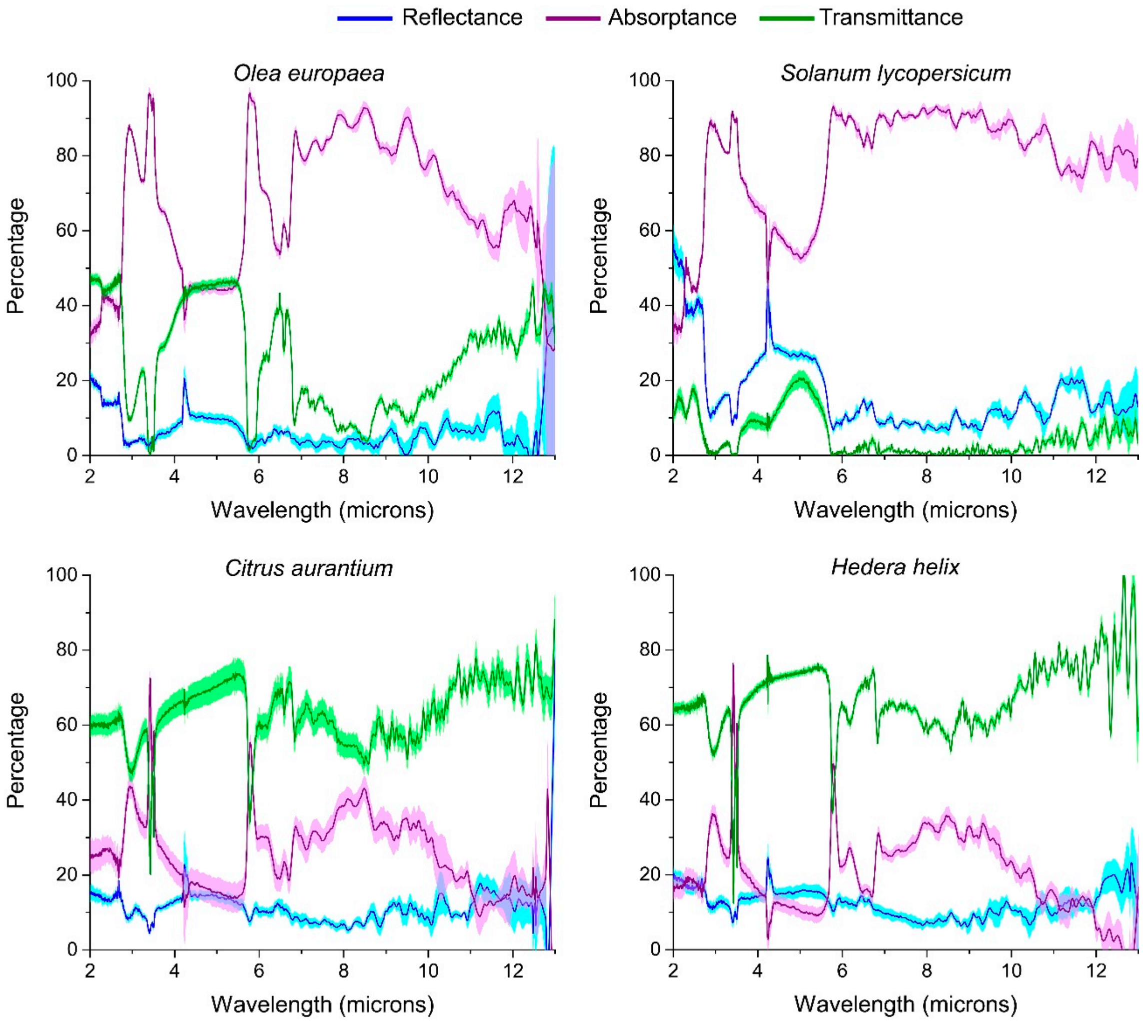


Figure S1. Transmittance, reflectance and absorptance spectra of isolated cuticles from different species in the Middle infrared region of the spectrum. Samples were measured from the inner side of the cuticle. *O. europaea*, *S. lycopersicum* and *V. vinifera* fruit cuticles. *H. helix* and *C. aurantium* adaxial leaf cuticles. 4-6 biological replicates per species were studied.

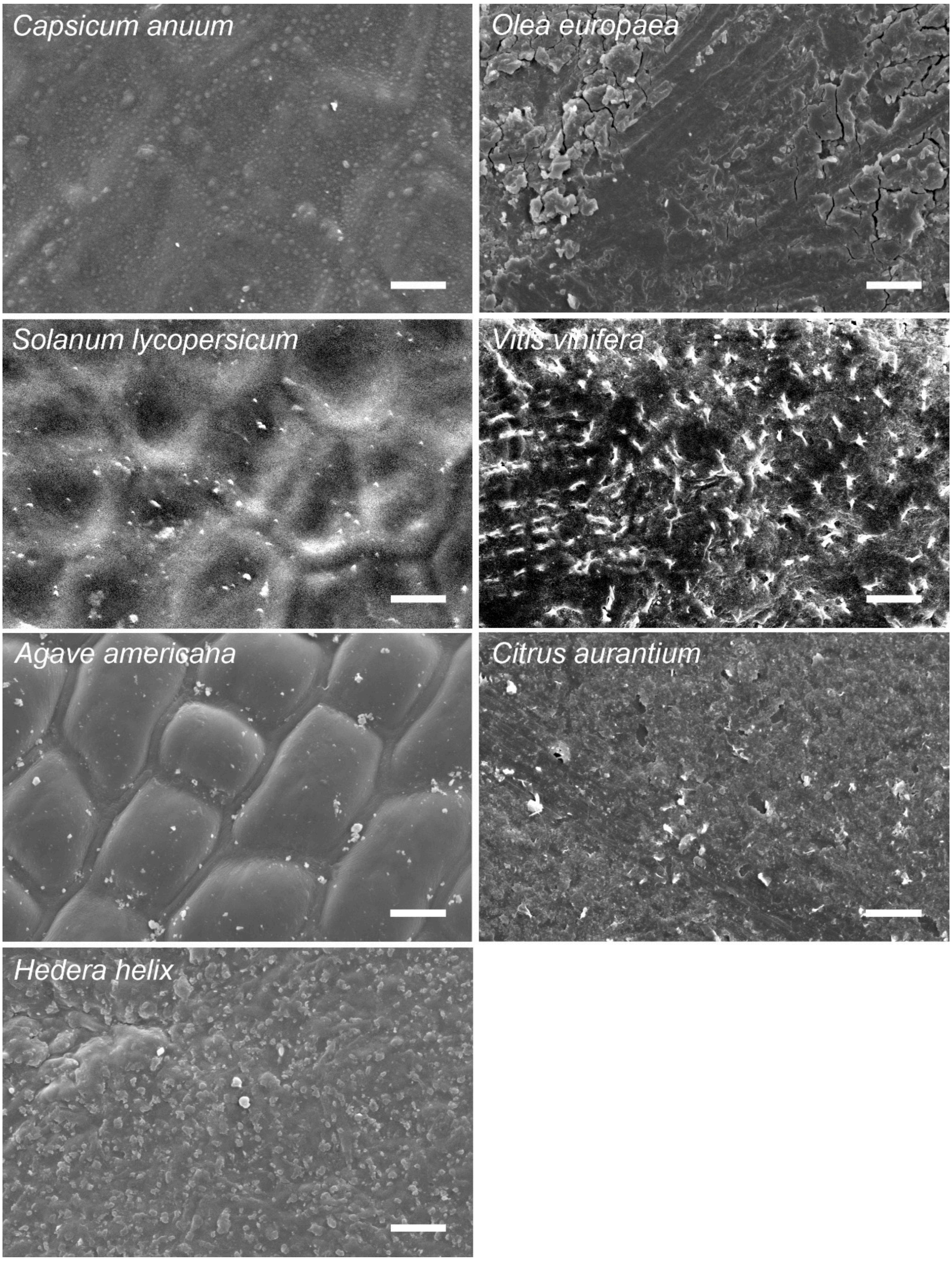


Figure S2. Scanning electron micrographs of the outer surface of fruits (*C. annuum*, *O. europaea*, *S. lycopersicum* and *V. vinifera*) and adaxial leaves (*A. americana*, *C. aurantium* and *H. helix*). Bars 20 µm.

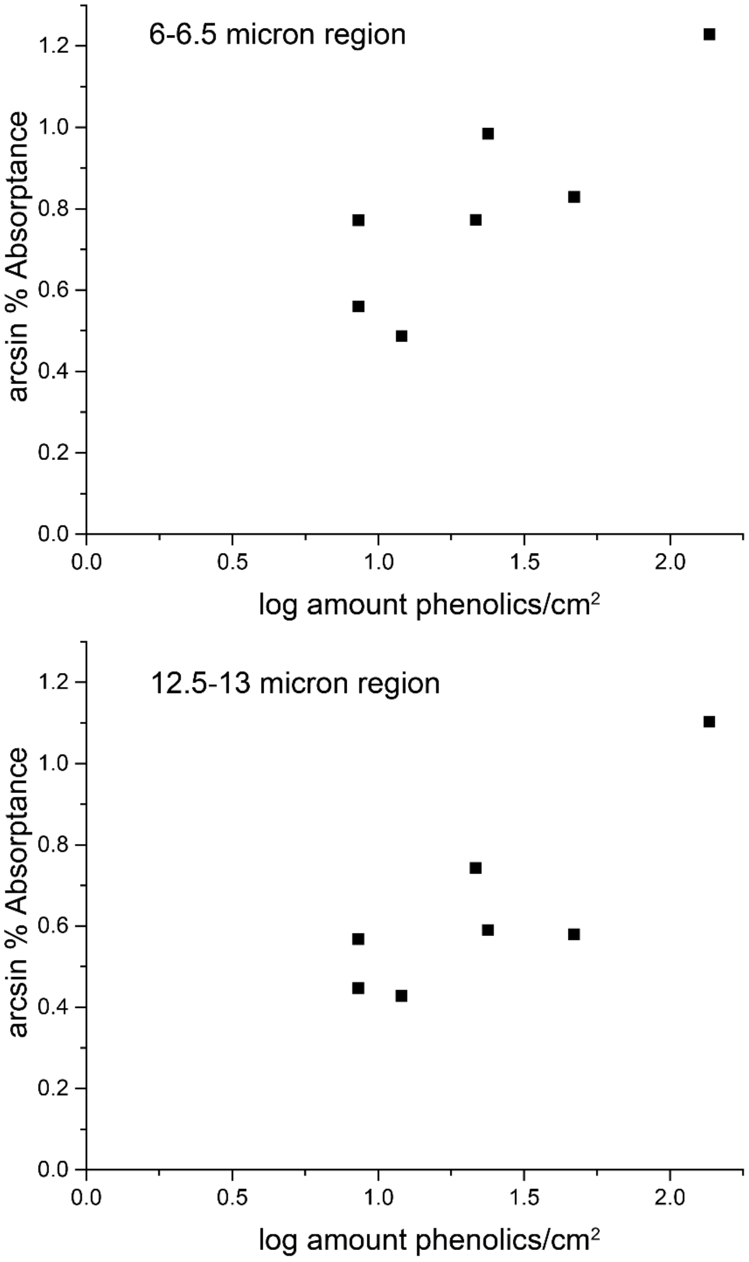


Figure S3. Relationship between transformed data for amount of cuticle phenolics and average absorptance within two spectral regions for the different species studied.

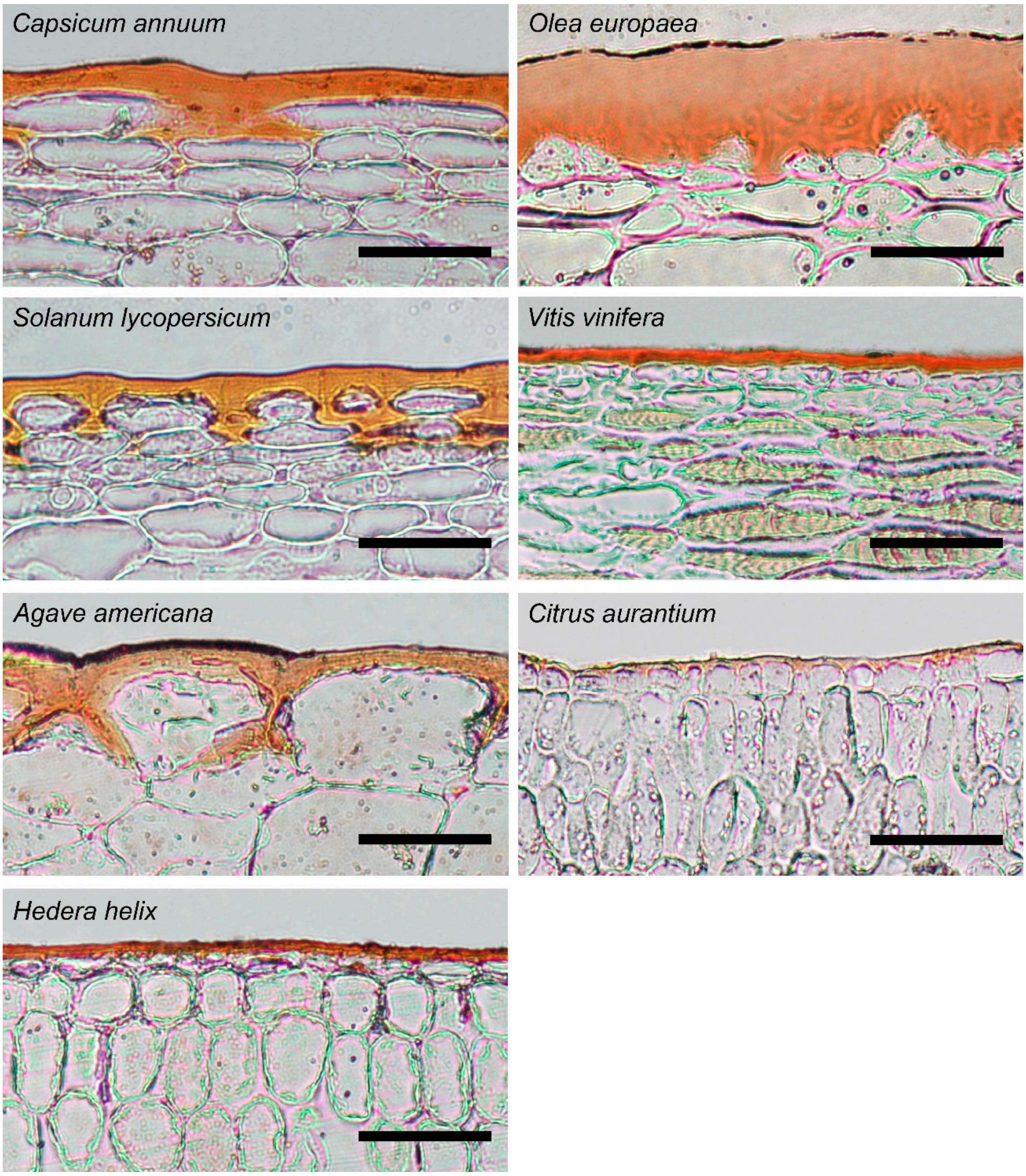


Figure S4. Photomicrographs of fruits and leaves cross sections from different species. Cuticle was stained with Sudan IV for visualization purposes. Fruit epidermises: *C. annuum*, *O. europaea*, *S. lycopersicum* and *V. vinifera*. Leaf adaxial epidermises: *A. americana*, *C. aurantium* and *H. helix*. Bars 50 µm.
